# Health-related quality of life associated with diabetic retinopathy in patients at a public primary care service in southern Brazil

**DOI:** 10.20945/2359-3997000000223

**Published:** 2020-03-30

**Authors:** Ângela Jornada Ben, Camila Furtado de Souza, Franciele Locatelli, Ana Paula Oliveira Rosses, Adriana Szortika, Aline Lutz de Araujo, Gabriela de Carvalho, Daniel Lavinsky, Jeruza Lavanholi Neyeloff, Cristina Rolim Neumann

**Affiliations:** 1 Faculty of Science Vrije Universiteit Amsterdam Amsterdam Public Health Research Institute Netherlands Department of Health Sciences, Faculty of Science, Vrije Universiteit Amsterdam, Amsterdam Public Health Research Institute, the Netherlands; 2 Faculdade de Medicina Universidade do Vale do Taquari Lajeado RS Brasil Faculdade de Medicina, Universidade do Vale do Taquari, Lajeado, RS, Brasil; 3 Faculdade de Ciências Médicas Universidade Federal do Rio Grande do Sul Porto Alegre RS Brasil Faculdade de Ciências Médicas, Universidade Federal do Rio Grande do Sul, Porto Alegre, RS, Brasil; 4 Departamento de Medicina Social Universidade Federal de Pelotas Pelotas RS Brasil Departamento de Medicina Social, Universidade Federal de Pelotas, Pelotas, RS, Brasil; 5 Serviço de Oftalmologia Hospital Moinhos de Vento Porto Alegre RS Brasil Serviço de Oftalmologia, Hospital Moinhos de Vento, Porto Alegre, RS, Brasil; 6 TelessaúdeRS Universidade Federal do Rio Grande do Sul Porto Alegre RS Brasil TelessaúdeRS, Universidade Federal do Rio Grande do Sul, Porto Alegre, RS, Brasil; 7 Hospital de Clínicas de Porto Alegre Porto Alegre RS Brazil Hospital de Clínicas de Porto Alegre, Porto Alegre, RS, Brazil; 8 Programa de Pós-Graduação em Epidemiologia Universidade Federal do Rio Grande do Sul Porto Alegre Brasil Programa de Pós-Graduação em Epidemiologia, Universidade Federal do Rio Grande do Sul, Porto Alegre, Brasil

**Keywords:** EQ-5D, health-related quality of life, utility values, diabetic retinopathy

## Abstract

**Objective:**

This study aimed to establish the utility values of different health states associated with diabetic retinopathy in a Brazilian sample to provide input to model-based economic evaluations.

**Subjects and methods:**

This cross-sectional study was performed in a sample of patients with type 2 diabetes mellitus (T2D) who underwent teleophthalmology screening at a primary care service from 2014 to 2016. Five diabetic retinopathy health states were defined: absent, non-sight-threatening, sight-threatening, and bilateral blindness. Utility values were estimated using the Brazilian EuroQol five dimensions (EQ-5D) tariffs. Descriptive statistics were calculated. Analysis of covariance was performed to adjust the utility values for potential confounders.

**Results:**

The study included 206 patients. The mean (± standard deviation [SD]) utility value was 0.765 ± 0.19 (95% confidence interval [CI], 0.740–0.790). The adjusted mean utility value was 0.748 (95% CI, 0.698–0.798) in patients without diabetic retinopathy, 0.752 (95% CI, 0.679–0.825) in those with non-sight-threatening state, 0.628 (95% CI, 0.521–0.736) in those with sight-threatening state, and 0.355 (95% CI, 0.105–0.606) in those with bilateral blindness. A significant utility decrement was found between patients without diabetic retinopathy and those with a sight-threatening health state (0.748 vs. 0.628, respectively, p = 0.04).

**Conclusions:**

The findings suggest that a later diabetic retinopathy health state is associated with a decrement in utility value compared with the absence of retinopathy in patients with T2D. The results may be useful as preliminary input to model-based economic evaluations. Further research is needed to investigate the impact of diabetic retinopathy on health-related quality of life in a sample more representative of the Brazilian population.

## INTRODUCTION

Diabetic retinopathy (DR) is one of the leading causes of preventable visual impairment and blindness worldwide, despite existing accurate diagnostic technologies and effective treatments (1,2). It is usually asymptomatic until late stages and could lead to a sudden visual loss affecting the individual’s functional capabilities (e.g., mobility, independence, self-care, and ability to perform daily activities such as work and leisure) (3). In Brazil, DR accounts for approximately 25% of all years lived with disability from diabetes mellitus (4).

The impact of DR on quality of life (also referred to as health-related quality of life [HRQoL]) has been reported in several countries (5,6). The results suggest that DR severity has a significant negative impact on HRQoL among patients with diabetes. However, this finding is not consistent across studies (5). HRQoL is a complex construct involving an individual’s perception of his or her health state (including physical, mental, and social domains) (7). Sociocultural differences may influence HRQoL perception (8). Therefore, it is particularly important to provide information on the DR impact on HRQoL considering the social context (9).

The EuroQol five dimensions (EQ-5D) (10) is the most commonly used generic preference-based measure of HRQoL (other measures include the SF-36 and the HUI-3) (11). It encompasses five health dimensions (mobility, self-care, usual activities, pain/discomfort, and anxiety/depression), all of which contain three severity levels, resulting in 234 possible health states. The health states can be converted into utility values (with a single value representing an individual’s preferences for a given health state) based on preferences of the general population (also referred to as tariffs). Utility values range from 1 (equaling full health) to 0 (equaling death). Negative values may also occur indicating that a person’s health state is worse than death (12). The obtained utility values can be used to calculate the quality-adjusted life-years (QALY) measure by multiplying the values by the amount of time spent in a specific health state (12). Many national guidelines (such as those from Brazil and the UK) (13,14) recommend using QALYs in economic evaluations to compare benefits from health technologies (12).

To the best of our knowledge, no study has been conducted using Brazilian EQ-5D tariffs to describe utility values according to DR health states. Therefore, this study aimed to establish the utility values for different health states associated with DR in a Brazilian sample to provide input to model-based economic evaluations, and to explore potential differences in HRQoL among DR health states.

## SUBJECTS AND METHODS

### Study design and population

This was a cross-sectional study including a convenience sample of patients with type 2 diabetes mellitus (T2D) who underwent teleophthalmology screening at a public primary care service in Southern Brazil from 2014 to 2016. Patients with T2D who were registered at the service were invited to participate by phone calls or were referred by the service’s family physicians for screening. Individuals with T2D who were older than 18 years were included. Patients were excluded if they had type 1 diabetes (T1D; n = 5, 2%), cognition problems (n = 0), blindness due to a disease other than T2D (n = 1, 0.4%), and unreadable retinal photographs due to lens opacity (n = 20, 8.6%). Patients with T1D were not included because of the low prevalence of this disease in primary care. Prior to the measurements, study requirements were explained to the patients by one of the three trained family physicians performing the teleophthalmology screening (15). Patients who agreed to participate provided written informed consent. A legal guardian signed the written informed consent in case of blindness. A sample size of 126 patients was required to detect a difference of 0.1 in mean utility value between two DR health states with an α of 0.05 and power of β = 80%.

The study was approved by the Ethics Committee of the *Hospital de Clínicas de Porto Alegre*.

### Teleophthalmology screening

Retinal photographs were taken by the aforementioned trained family physicians. Images of two fields of each eye were captured by using the Canon CR-2 Digital Retinal (Canon U.S.A., Inc., Melville, NY, USA). Retinal photographs were remotely evaluated and classified by two ophthalmologists of the teleophthalmology screening based on the International Clinical Diabetic Retinopathy and Diabetic Macular Edema Disease Severity Scale (16). More details about the teleophthalmology screening training and work process have been described by other authors (15).

### Diabetic retinopathy health states

Five DR health states were defined based on economic evaluation models previously published in the literature (17-19): absent (NoDR), non-sight-threatening (Non-STDR), sight-threatening (STDR), and bilateral blindness (BB). The Non-STDR health state included mild and moderate nonproliferative DR. The STDR health state included severe nonproliferative, proliferative DR, and clinically significant macular edema. The categorization as Non-STDR and STDR was based on the worse eye. Patients were asked to report previously diagnosed eye conditions. Patients reporting a complete vision loss in both eyes due to T2D and presenting retinographic findings suggestive of vision loss due to DR were classified as BB.

### Measure of Health-Related Quality of Life – Utility values

The EQ-5D is a standardized, generic preference-based measure of HRQoL developed by the EuroQol Group (10). The three-level version of the EQ-5D consists of two pages: the EQ-5D descriptive system and the EQ visual analogue scale (EQVAS). The descriptive system comprises five HRQoL dimensions (mobility, self-care, usual activities, pain/discomfort, and anxiety/depression). Each dimension has three severity levels (no problems, some problems, and extreme problems). The EQVAS records the patient’s self-rated health on a vertical visual analogue scale ranging from 0 to 100, where the endpoints are labeled “best imaginable health state” and “worst imaginable health state” (10). The visual analogue scale can be used as a quantitative measure of health outcome that reflects the patient’s own judgment (10). The patients completed the questionnaire before the retinal photographs were taken. The three-level version of the EQ-5D has been validated in Portuguese, and Brazilian tariffs have been published (20).

### Description of variables

Demographic and clinical variables of interest for descriptive analysis were collected from electronic medical records: age (years), sex, self-reported skin color (white or non-white), education level (no/primary education, secondary education, higher education), diabetes duration in years, diabetes treatment, glycated hemoglobin (HbA1c), diagnosis of hypertension, creatinine, albuminuria, dialysis, low-density lipoprotein cholesterol (LDL), triglycerides (TG), high-density lipoprotein cholesterol (HDL), presence of foot ulcers or lower-extremity amputation, previous coronary heart disease, stroke, ophthalmic diseases, and other self-reported comorbidities. We collected the most recent laboratory results available in the patients’ electronic medical record within 12 months prior to the screening. Diabetes control was defined as an HbA1c level ≤ 7.0% (21). Hypertension was defined as current antihypertensive therapy and/or hypertension diagnosis reported in the medical record. Levels of systolic and diastolic blood pressure below 140 mmHg and 90 mmHg, respectively, were classified as controlled hypertension. Chronic kidney disease was defined as any abnormal albuminuria in a spot urine sample (≥ 17 mg/L or 20–200 mg/g Cr) or a glomerular filtration rate < 90 mL/min/1.73 m^2^ (21). Dyslipidemia was defined as values of LDL cholesterol ≥ 160 mg/dL, or TG ≥ 150 mg/dL, or HDL < 40 mg/dL (men) and < 50 mg/dL (women) (21). Dialysis, lower-extremity amputation, foot ulcers, coronary heart disease, stroke, and ophthalmic disease were inquired through direct questions. Ophthalmic diseases included refractive errors, cataract, glaucoma, ocular toxoplasmosis, and other self-reported ocular conditions. Age-related macular degeneration was assessed by two of the aforementioned ophthalmologists through digital retinal photographs and by patient self-report.

### Statistics analyses

Missing data related to demographic and clinical variables (214 [2.6%] out of 8026 values) were imputed by means of regression models. Descriptive statistics were calculated using pooled data from the 10 imputed data sets. Means ± standard deviations (SD) were used to describe normally distributed variables, and medians and interquartile range (IQR) were used for nonparametric variables. The normality of variables was evaluated by histogram graphs and the Kolmogorov-Smirnov test.

The utility values for different health states associated with diabetic retinopathy were assessed with adjustment for potential confounders using analysis of covariance (ANCOVA). The variables included in the adjusted analysis were selected from the two following sources: a) from a theoretical model based on the current literature, those variables associated with HRQoL, such as age, sex, other comorbidities, ophthalmic diseases, and macrovascular and microvascular complications (22,23) and b) from a previous univariate analysis, those variables found to be associated with utility values (*p *≤ 0.05). Diabetes duration, HbA1c, diabetes control, and type of treatment were not included in the adjusted analysis because they are usually not associated with HRQoL, despite their strong association with DR (21,24,25). Additional adjusted analysis was performed after excluding the cases of BB because this group was very small (two cases).

We opted to perform ANCOVA because there was homogeneity of variances in utility values at each level of DR (Levene’s test, *p *= 0.27) and the residuals followed an approximately normal distribution. For the adjusted analysis, we grouped chronic kidney disease, foot ulcers, and lower-extremity amputation into a single variable named “microvascular complications”. Coronary heart disease and stroke were grouped into a variable named “macrovascular complications”. Cataract, glaucoma, ocular toxoplasmosis, age-related macular degeneration, and other self-reported ocular diseases were grouped into a variable named “ophthalmic diseases”. A variable named “other comorbidities” included other self-reported diseases not included in the three previous variables, such as cancer and rheumatologic and dermatologic disorders. Additional interaction analysis was undertaken considering all possible interactions between variables included in the adjusted analysis. IBM SPSS Statistics version 24.0 was used to perform all analyses.

## RESULTS

We included 206 out of the 232 patients who underwent the teleophthalmology screening. The mean age of the patients included was 63.5 ± 10.6 years, 60.7% (n = 125) were female, 85% (n = 175) were of white ethnicity, and 50.5% (n = 104) had secondary education. The patients included in the study had a statistically significantly higher mean utility value compared with those who were excluded due to unreadable retinal photographs (0.765 ± 0.19 *vs.* 0.636 ± 0.18, respectively, *p*=0.004). However, there were no significant differences between included and excluded patients regarding HbA1c (7.5% *vs* 7.0%, respectively, *p *= 0.13), diabetes control (68.4% *vs* 71.1%, respectively, *p *= 0.49), and diabetes duration (8.7 *vs.* 8.2 years, respectively, *p *= 0.71).

The overall prevalence of DR was 23.8% (n = 49). In all, 15.5% (n = 32) of the patients had Non-STDR, 7.3% (n=15) had STDR, and 1% (n = 2) had BB (Table 1). The percentage of patients reporting full health was 25.7% (n = 53). The mean utility was 0.773 ± 0.17 in patients with NoDR and 0.739 ± 0.24 in those with DR. Patients with DR and no BB presented a mean utility of 0.755 ± 0.23, whereas those with BB presented a mean utility of 0.356 ± 0.21. Appendices 1 and 2 provide a more detailed description of the sample regarding the five EQ-5D dimensions of quality of life according to DR health states.

**Table 1:** Demographic and clinical characteristics of the sample by diabetic retinopathy (DR) health states

Characteristics	NoDR	Non-STDR	STDR	BB
n (%)	157 (76.2)	32 (15.5)	15 (7.3)	2 (1.0)
Age, mean (SD)	63.9 (11.0)	60.0 (8.8)	66.5 (9.1)	63.3 (6.1)
Female, n (%)	104 (66.2)	12 (37.5)	7 (46.7)	2 (100)
White, n (%)	135 (86.0)	23 (71.9)	15 (100)	2 (100)
Education level, n (%)				
No/primary education	44 (28.0)	6 (18.8)	1 (6.7)	1 (50)
Secondary education	76 (48.4)	17 (53.1)	10 (66.7)	1 (50)
Higher education	37 (23.6)	9 (28.1)	4 (26.7)	0
HbA1c, mean value in % (SD)	7.5 (1.9)	8.4 (2.1)	8.5 (1.5)	8.8 (3.1)
Controlled DM, n (%)	88 (56.1)	11 (34.4)	3 (20.0)	1 (50.0)
DM duration in years, median (IQR)	5 (2-10)	6 (3-13)	16 (5-25)	18 (10-26)
Insulin treatment, n (%)	28 (17.8)	15 (46.9)	12 (80.0)	2 (100)
Controlled hypertension, n (%)	89 (69.0)	15 (57.7)	9 (69.2)	1 (50.0)
Dyslipidemia, n (%)	112 (71.3)	23 (71.9)	11 (73.3)	2 (100)
Macrovascular complications, n (%)	36 (22.9)	11 (34.4)	5 (33.3)	0
Microvascular complications, n (%)	100 (63.7)	21 (65.6)	14 (93.3)	2 (100)
Ophthalmic diseases, n (%)	34 (21.7)	3 (9.4)	2 (13.3)	0
Other comorbidities, n (%)	38 (24.2)	3 (9.4)	6 (40.0)	1 (50.0)
EQ-5D utility, mean (SD)	0.773 (0.17)	0.801 (0.21)	0.658 (0.25)	0.356 (0.21)
EQVAS, median (IQR)	80 (60-90)	75 (70-80)	70 (50-80)	65 (50-80)

NoDR: absence of diabetic retinopathy; Non-STDR: non-sight-threatening diabetic retinopathy; STDR: sight-threatening diabetic retinopathy; BB: bilateral blindness; DM: diabetes mellitus; SD: standard deviation; IQR: interquartile range; Macrovascular complications: coronary heart disease and stroke; Microvascular complications: chronic kidney disease (n = 136), foot ulcer (n = 2), and lower-extremity amputation (n = 1). Ophthalmic diseases: cataract, glaucoma, and age-related macular degeneration; EQVAS: visual analogue scale EQ-5D.

Table 2 shows the utility values for the different health states related to DR with and without adjustment for potential confounders. The mean utility value of the various DR health states decreased after adjustment. The adjusted mean utility was 0.748 (95% CI, 0.698 – 0.798) for a NoDR health state, 0.752 (95% CI, 0.679 – 0.825) for Non-STDR, 0.628 (95% CI, 0.521 – 0.736) for STDR, and 0.355 (95% CI, 0.105 – 0.606) for BB. The adjusted analysis performed after excluding the two cases of BB showed a statistically significant utility decrement between patients at NoDR and STDR health sates (0.748 *vs.* 0.628, respectively, *p *= 0.04). No significant differences were found between NoDR and non-STDR health states (0.748 *vs.* 0.752, respectively, *p *= 1.0) and between non-STDR and STDR health states (0.752 *vs*. 0.628, respectively, *p *= 0.07).

**Table 2:** Mean EuroQol five dimensions (EQ-5D) utility values by variables in unadjusted and adjusted analysis

	Mean (95% CI)	p value	Adjusted mean* (95% CI)	F	*p* value
Sex					
Female	0.727 (0.695 – 0.760)	0.001	0.572 (0.487 – 0.658)	14475	0.001
Male	0.824 (0.783 – 0.864)		0.671 (0.581 – 0.759)		
Skin color					
White	0.753 (0.724 – 0.781)	0.020	0.601 (0.524 – 0.678)	1290	0.258
2Non-white	0.836 (0.772 – 0.900)		0.641 (0.539 – 0.744)		
DM treatment					
Diet	0.779 (0.693 – 0.866)	0.283	NA	NA	NA
Oral medications	0.778 (0.748 – 0.808)				
Insulin	0.731 (0.671 – 0.791)				
Controlled DM					
Yes	0.774 (0.743 – 0.806)	0.359	NA	NA	NA
No	0.756 (0.714 – 0.798)				
Controlled hypertension					
Yes	0.756 (0.723 – 0.789)	0.549	NA	NA	NA
No	0.774 (0.771 – 0.832)				
Dyslipidemia					
Yes	0.751 (0.720 – 0.782)	0.123	NA	NA	NA
No	0.801 (0.752 – 0.850)				
Macrovascular complications					
Yes	0.687 (0.629 – 0.746)	0.001	0.563 (0.468 – 0.657)	15786	0.001
No	0.791 (0.764 – 0.819)		0.679 (0.598 – 0.761)		
Microvascular complications					
Yes	0.745 (0.711 – 0.780)	0.060		0.872	0.352
No	0.804 (0.767 – 0.842)				
Other comorbidities					
Yes	0.709 (0.653 – 0.765)	0.020	0.608 (0.529 – 0.687)	1757	0.187
No	0.782 (0.753 – 0.811)		0.634 (0.538 – 0.731)		
Ophthalmic diseases					
Yes	0.714 (0.659 – 0.769)	0.060		2188	0.141
No	0.777 (0.748 – 0.806)				
DR health states					
NoDR	0.773 (0.746 – 0.800)	0.001	0.748 (0.698 – 0.798)**	5041	0.002
Non-STDR	0.801 (0.726 – 0.875)		0.752 (0.679 – 0.825)**		
STDR	0.658 (0.521 – 0.794)		0.628 (0.521 – 0.736)		
BB	0.356 (0.061 – 0.651)		0.355 (0.105 – 0.606)**		

SD: standard deviation; NA: not applicable, variables not included in the adjusted analysis; DM: diabetes mellitus; DR: diabetic retinopathy; NoDR: absence of diabetic retinopathy; Non-STDR: non-sight-threatening diabetic retinopathy; STDR: sight-threatening diabetic retinopathy; BB: bilateral blindness. *Analysis adjusted by age, sex, skin color, other comorbidities, macrovascular and microvascular complications, and ophthalmic diseases. R square = 0.242; Adjusted R square = 0.202. **Bonferroni test: NoDR and BB (p = 0.01); Non-STDR and BB (p = 0.01).

**Figure 1:**
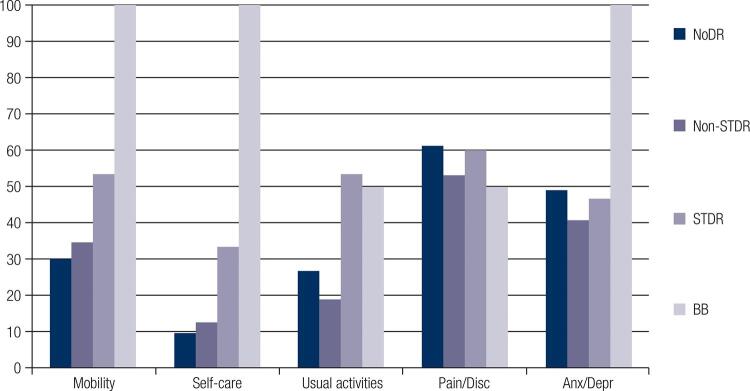
Plots showing mean utility values by diabetic retinopathy (DR) health states stratified by sex, other comorbidities, ophthalmic diseases, and macrovascular and microvascular complications. A statistically significant interaction was observed between other comorbidities and DR health states. NoDR: absence of diabetic retinopathy; Non-STDR: non-sight-threatening diabetic retinopathy; STDR: sight-threatening diabetic retinopathy; BB: bilateral blindness.

The interaction analysis showed statistically significant interactions between DR health states and other comorbidities (F_3,66 _= 3679, *p *= 0.01); between sex, skin color, and DR health states (F_1,66_ = 6020, *p *= 0.01); between other comorbidities, macrovascular complications, and DR health states (F_1,66 _= 8596, *p *< 0.001); and between skin color and microvascular complications (F_1,66 _= 3974, *p *= 0.05). The interaction between DR health states and the variables included in the adjusted analysis is presented in Figure 1.

## DISCUSSION

This study established the utility values for different health states associated with DR in a sample of patients with T2D undergoing teleophthalmology screening at a public primary care service in Southern Brazil. The results suggest that a later DR health state is associated with a significant decrement in HRQoL compared to the absence of retinopathy in patients with T2D. Additional interaction analysis suggests that the utility values for different health states associated with DR may depend on a combination of DR with other factors such as sex, skin color, other comorbidities, and macrovascular complications. Additional research is needed to further establish this association.

Research exploring the utility values of different health states associated with DR has provided mixed results (26-29). Heintz and cols. (27) found no difference in mean utility values across various levels of DR severity in a sample of Swedish patients with T1D and T2D. Fenwick and cols. also found no differences in a sample of Australian patients with T1D and T2D (28). Our study differs from both these studies because it was based on a different population (comprising only individuals with T2D) and included different variables in the adjusted analysis (i.e., did not include variables strongly associated with DR, such as diabetes duration and HbA1c, which are usually not directly related to HRQoL). Similar to our results, Polack and cols. found that late DR health states were associated with a lower mean utility value compared with the absence of DR in a sample of patients with T2D in India (29). Lloyd and cols. found significant utility value decrements associated with lower visual acuity in a sample of T1D and T2D patients in the UK (26). The present study also found no HRQoL differences in early DR health states (i.e., between patients without DR and Non-STDR), which is in agreement with other studies suggesting that early DR health states are unlikely to be strongly correlated with any of the dimensions of HRQoL (27,28,30).

This study has a number of limitations that need to be discussed. First, the EQ-5D may not be sensitive enough to detect small differences in HRQoL during early DR health states (27). The validity of EQ-5D compared to other generic preference-based measures of HRQoL (e.g., HUI-3) regarding DR progression is controversial (26,27). Researchers have proposed adding bolt-ons to expand EQ-5D descriptive systems considering visual symptoms however, this is still under investigation (31).

Second, the convenience sample only allowed us to assess patients registered at a primary care service, thus potentially reducing the generalizability of the findings. Therefore, we would advise researchers to only use these numbers as preliminary input to model-based economic evaluations. Nonetheless, it is noteworthy that the mean overall utility value reported in our study (0.76 ± 0.19) was similar to values found in developed countries, such as the UK (0.77 ± 0.27) (32) and the Netherlands (0.74 ± 0.27) (33).

Third, this study did not directly assess visual acuity, which is known to be associated with lower HRQoL in late DR health sates (34,35). Consequently, we had to rely on DR diagnosis/classification by image without adjustment for the visual acuity potential confounder. Nevertheless, to be able to populate model-based economic evaluations, the utility values should be classified according to DR health states instead of visual acuity (27).

Fourth, patients with unreadable photographs were excluded from the study, which may have biased the results due to selective patient exclusion. However, the lower HRQoL presented by the excluded patients compared with those included in the study may be related to the lens opacity instead of DR, since there was no difference regarding diabetes control and duration between them.

Bearing in mind that HRQoL could be different across country populations and that one of the main outcomes of economic evaluations (i.e., QALY gained) relies on utility values, this study was the first attempt to describe HRQoL associated with DR health states in a Brazilian primary care setting based on general population preferences. These results may be useful as preliminary input to model-based economic evaluations. Further research is needed to investigate the impact of DR progression on HRQoL in a representative sample of the Brazilian population.

In conclusion, this study established the utility values for different health states associated with DR in a Southern Brazilian sample of patients with T2D undergoing teleophthalmology screening at a public primary care service. The results suggest that a late DR health state is associated with decrements in HRQoL. The findings may be useful as preliminary input to model-based economic evaluations.

**Appendix 1:** Frequency and proportion of EuroQol five dimensions (EQ-5D) by diabetic retinopathy (DR) health states

		NoDR n (%)	Non-STDR n (%)	STDR n (%)	BB n (%)	p value*
		n =157	n =32	n =15	n = 2	
Mobility	No problems	110 (70.1)	21 (65.6)	7 (46.7)	0	0.055
	Some problems	46 (29.3)	11 (34.4)	7 (46.7)	2 (100)	
	Extreme problems	1 (0.6)	0	1 (6.7)	0	
Self-care	No problems	142 (90.4)	28 (87.5)	10 (66.7)	0	0.01
	Some problems	13 (8.3)	4 (12.5)	4 (2.7)	0	
	Extreme problems	2 (1.3)	0	1 (6.7)	2 (100)	
Usual activities	No problems	115 (73.2)	26 (81.3)	7 (46.7)	1 (50.0)	0.01
	Some problems	39 (24.8)	5 (15.6)	6 (40.0)	0	
	Extreme problems	3 (1.9)	1 (3.1)	2 (13.3)	1 (50.0)	
Pain/discomfort	No problems	61 (38.8)	15 (46.9)	6 (40.0)	1 (50.0)	0.97
	Some problems	91 (58.0)	16 (50.0)	8 (53.3)	1 (50.0)	
	Extreme problems	5 (3.2)	1 (3.1)	1 (6.7)	0	
Anxiety/depression	No problems	80 (51.0)	19 (59.4)	8 (53.3)	0	0.26
	Some problems	67 (42.7)	10 (31.3)	6 (40.0)	1 (50.0)	
	Extreme problems	10 (6.4)	3 (9.4)	1 (6.7)	1 (50.0)	

NoDR: absence of diabetic retinopathy; Non-STDR: non-sight-threatening diabetic retinopathy; STDR: sight-threatening diabetic retinopathy; BB: bilateral blindness.

* Chi-square test.

**Appendix 2:**
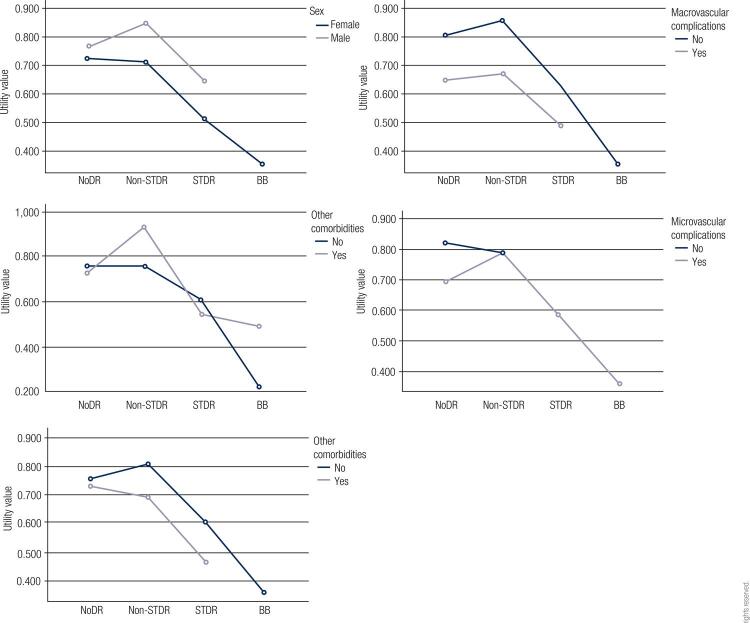
Percentage of reported problems by dimension of the EuroQol five dimensions (EQ-5D) according to diabetic retinopathy (DR) health states. NoDR: absence of diabetic retinopathy; Non-STDR: non-sight-threatening diabetic retinopathy; STDR: sight-threatening diabetic retinopathy; BB: bilateral blindness; Disc: Discomfort. Anx/Depr: Anxiety/Depression.
